# 基于微流控技术的外泌体分离分析及临床应用

**DOI:** 10.3724/SP.J.1123.2024.10032

**Published:** 2025-05-08

**Authors:** Yuhang XING, Xiangshan REN, Donghao LI, Lu LIU

**Affiliations:** 1.延边大学理学院化学系, 长白山天然药物研究教育部重点实验室, 吉林 延吉 133000; 1. Department of Chemistry, College of Science, Yanbian University, Key Laboratory of Natural Medicine Research in Changbaishan, Ministry of Education, Yanji 133000, China; 2.延边大学医学院, 吉林 延吉 133000; 2. Yanbian University Medical College, Yanji 133000, China

**Keywords:** 微流控, 外泌体, 分离分析, 疾病诊断及治疗, microfluidics, exosomes, separation analysis, disease diagnosis and treatment

## Abstract

外泌体是由细胞分泌的纳米级囊泡,其中富含脂质、蛋白质、核酸等多种生物功能分子。外泌体在生物体内的信息传递、疾病机理研究及诊断等方面扮演着类似“物联网、互联网”的关键角色。然而,受限于外泌体来源、尺寸、内含物以及功能的异质性,外泌体分离分析仍然面临诸多挑战。传统外泌体分离分析方法包括超速离心、尺寸排阻和免疫共沉淀等,但这些方法普遍存在产量低、纯度低等问题限制了其进一步的临床应用。微流控技术因其微型化、高通量、自动化和集成化等特点为复杂生物样本的高效分离分析提供了强有力的工具。基于此,本文系统总结了基于微流控技术的外泌体分离和分析方法。首先,讨论了基于外泌体的尺寸、电荷、表面官能团等物化性质结合微流控技术分离外泌体及外泌体亚群的分离方法;其次,分析了基于微流控平台的外泌体检测手段,包括外泌体群体分析方法和单个外泌体分析方法;最后,探讨了微流控技术在外泌体临床应用中的前景,并在疾病诊断和治疗方面进行了展望。微流控技术有望突破传统研究中的分离分析瓶颈,为癌症等重大疾病的精准诊断及治疗提供新技术、新平台。

## Abstract (en)

Exosomes are cell-secreted nanoscale vesicles 30-150 nm in size and encompass a diverse array of biomolecules, including lipids, proteins, and nucleic acids. Exosomes play pivotal roles during the intercellular exchange of materials and information, and are closely associated with the onset and progression of a variety of diseases. Therefore, comprehensively investigating exosomes is very important in terms of disease diagnosis and treatment. However, exosomes are genetically heterogeneous and are composed of different materials. Additionally, exosome-size and packing-specific-biomarker heterogeneities result in biofunction diversity. Moreover, isolating and analyzing exosomes is highly challenging owing to their small sizes and heterogeneities. Accordingly, effective separation methods and analytical techniques for highly specifically and efficiently identifying exosomes are urgently needed in order to better understand their functionalities.

While separation and analysis is required to reveal exosome heterogeneity, the former is confronted by three primary challenges. Firstly, exosome heterogeneity (including heterogeneous marker expressions and size heterogeneity that results in heterogeneous functions) results in systems that are very difficult to separate. Secondly, the coexistence of non-vesicular contaminants (lipoprotein nanoparticles, soluble proteins, nucleic acids, etc.) and the complex matrix effects of body fluids also contribute to separation difficulties. Thirdly, enrichment is a highly challenging task owing to low exosome concentrations. Traditional methods, such as ultracentrifugation and size-exclusion chromatography, fall short in terms of their abilities to precisely separate and analyze exosomes. On the other hand, microfluidics has emerged as a robust tool for the efficient analysis of complex biological samples and is characterized by miniaturization, precise control, high throughput, automation, and integration. Firstly, the operability, integrability, and modifiability of a microfluidics system facilitate exosome separation and purification based on surface properties, size, charge, and polarity. Secondly, the use of a microfluidics approach, with its high throughput, low reagent consumption, and multichannel manipulability, greatly facilitates preparing exosomes and enhancing their concentrations. Thirdly, microfluidics ensures that diverse separation methods are compatible with downstream analysis techniques.

Exosomes are highly heterogeneous; hence, they are classified by type and subpopulation (according to origin, size, molecular markers, functions, etc.). This paper first discusses microfluidics techniques for separating exosomes and examines various separation strategies grounded in the physicochemical properties of exosomes. We then analyze exosome detection methodologies that use microfluidics platforms and encompass traditional group-exosome analysis techniques and novel single-exosome analysis approaches. Finally, we discuss future clinical applications of microfluidics technology in exosome research, particularly its potential for diagnosing and treating diseases, thereby underscoring the applications value of microfluidics technology in the realm of personalized and precision medicine. Furthermore, cutting-edge microfluidics platforms offer novel perspectives for purifying and preparing EVs owing to precise fluid control, integration, miniaturization, and high-throughput characterization. EV populations, subpopulations, and single vesicles can be purified based on their physicochemical properties and microfluidics features. Comprehensive lab-on-a-chip methods are promising in terms of separating EVs based on traits, such as size, surface markers, and charge, and for obtaining highly pure EVs. Recycled EV samples can be prepared by controlling the high-throughput and multichannel capabilities of microfluidics approaches. The transition from bulk EV analysis to single-vesicle analysis provides opportunities to explore the heterogeneous nature of EVs, thereby augmenting their potential for disease diagnosis.

细胞外囊泡根据大小和来源可以分为3大类:外泌体、微囊泡和凋亡小体^[1,2]^。其中外泌体(尺寸30~150 nm)是细胞间信息传递的重要媒介^[3]^,具有运输货物^[4]^、传递信号等功能^[5]^。外泌体的形成和释放受到细胞状态、微环境和外部刺激等多种因素的影响^[6]^。外泌体的生成始于细胞膜的内吞作用,这一过程导致内体的形成,随后内体膜进一步向内凹陷,最终形成多泡体(multivesicular body, MVBs),多泡体与细胞膜融合将外泌体释放到细胞外空间,而部分未释放到细胞外的多泡体与溶酶体融合导致其内容物被降解^[7]^。细胞所分泌的外泌体具有脂质双层膜结构,因此可以有效地保护内容物不被降解,并且可以稳定地运输蛋白质、脂质、核酸等生物活性成分^[8⇓⇓⇓-12]^。在细胞膜两次内陷的过程中,细胞质中的内容物、跨膜蛋白和膜表面蛋白等被包裹在外泌体中^[13]^,因此外泌体具有丰富的生物学功能(见图1)^[14,15]^。

**图1:**
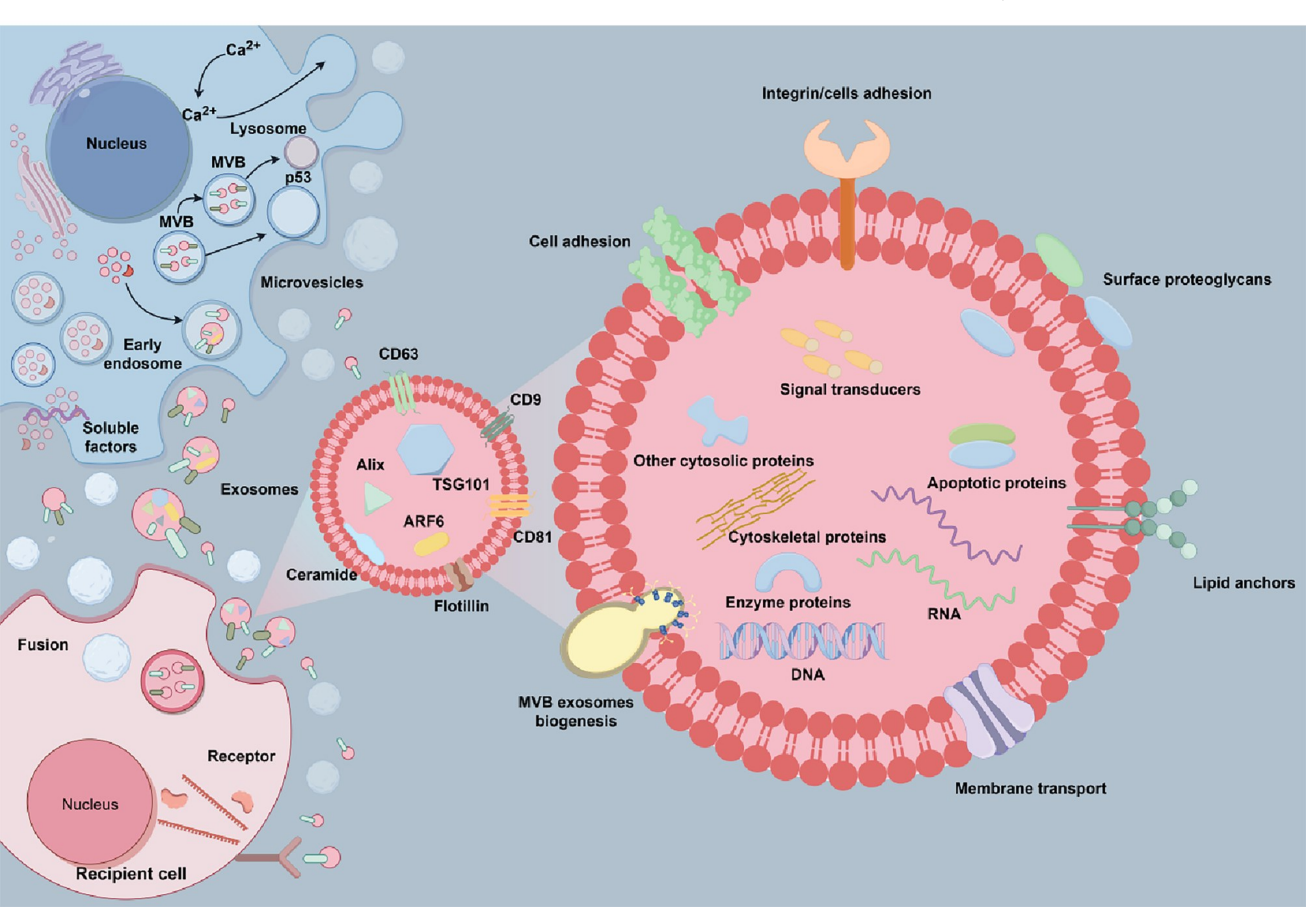
外泌体生物合成和分泌的细胞内途径(本图采用Figdraw绘制)

随着科学研究的不断深入,外泌体在临床诊断^[16,17]^、疾病生物标志物的检测^[18,19]^、精准治疗^[20]^等领域发展迅速。近年来,外泌体检测作为一种新兴的液体活检手段,可以在肿瘤细胞分泌的外泌体中获取到关于肿瘤的分子信息^[19,21]^,相比于组织活检,外泌体检测可以从有限的临床样品中获取到更准确的信息,可以作为新型肿瘤标志物来评估肿瘤分期和类型^[22]^。此外,近期研究发现外泌体中含有丰富的生长因子和细胞因子,可以作为细胞的营养来源^[23]^,有助于促进皮肤细胞的再生和修复^[24]^。

外泌体的异质性包括来源、尺寸、生物学及功能异质性。如何从复杂的细胞外基质或体液中实现异质性外泌体的无损、高效、在线分离、制备及检测是分析化学中亟待解决的关键科学问题^[25⇓-27]^。然而,外泌体体积小、基质复杂、尺寸与其他颗粒相似等特征严重干扰了其分离与分析^[28]^。目前常用的外泌体分离方法有超速离心法(ultracentrifugation, UC)^[29]^、尺寸排阻色谱法^[30]^、免疫捕获^[31,32]^等,但这些方法存在操作复杂、耗时费力、通量有限等缺陷,在疾病诊断及临床治疗等方面仍存在一定的局限性。UC作为外泌体制备的“金标准”,其所利用的巨大离心力可能会使外泌体的结构被破坏^[33⇓⇓-36]^,并且经过该方法制备的外泌体样品中存在大量的杂蛋白,导致外泌体纯度较低^[33,37]^,因此很难满足后续的生物学研究需求。

微流控技术因具有微型化、集成化、自动化等特征,为外泌体的分离和分析提供了新方法。本文基于外泌体的物化性质系统总结了微流控技术分离外泌体的3种策略,分别为基于外泌体尺寸及密度的分离技术、基于外泌体电荷的分离技术和基于外泌体免疫亲和的分离技术等,随后总结了外泌体的分析方法,包括基于微流控的外泌体群体分析方法和单个外泌体分析方法,最后总结了外泌体在诊断及治疗方面的临床应用。

## 1 基于微流控技术分离外泌体

基于微流控技术分离外泌体的策略包括以下3种:基于外泌体的尺寸和密度等的分离技术,包括膜分离、侧向位移、声场分离等;基于外泌体表面电荷的分离技术,包括凝胶电泳、毛细管电泳(CE)和自由流电泳(free flow electrophoresis, FFE)、电场流分离技术、介电泳(dielectrophoresis, DEP)技术等;基于外泌体的免疫亲和捕获包括抗体修饰及适配体修饰等,下面对这几种基于微流控分离外泌体方法进行详细介绍。

### 1.1 基于外泌体尺寸分离的微流控技术

为了实现外泌体的分离可以首先从其尺寸入手。体液等复杂基质中细胞尺寸一般在微米尺度,而蛋白质、核酸等尺寸仅为几个纳米,外泌体的尺寸分布为30~150 nm,同时外泌体的尺寸和其他细胞外囊泡相比也存在差异,因此可以利用尺寸特征将外泌体从复杂基质中纯化并制备出来。此外,值得注意的是外泌体本身存在尺寸异质性,不同尺寸的外泌体所携带的内含物不同导致生物功能的异质性,因此结合微流控技术的可操纵及集成化等特征精准分离出尺寸异质性外泌体是非常有必要的。

#### 1.1.1 膜分离技术

切向流分离(tangential flow filtration, TFF)是一种样品在压力的驱动下,根据过滤膜孔径大小进行物质分离的一种膜过滤过程。样品沿膜的表面以切向方向流动,这种方式有效减少了膜表面的沉积,可极大地提高样品的分离效率。Haraszti等^[38]^采用TFF技术分离外泌体(见图2a),以三维培养的干细胞细胞外囊泡(mesenchymal stem cells extracellular vesicles, MSC EVs)为例,该方法与金标准UC相比显著提高了的外泌体产量。但是该方法在分离外泌体过程中会有一定比例损失,影响外泌体的回收率。为了提升TFF的回收率,Hua等^[39]^设计了一种基于双切向流过滤的微流控芯片用于从生物液体中分离外泌体(见图2b)。通过微流控技术中的蛇形通道使外泌体在多孔膜上进行切向流动。实验结果表明通过该方法分离的外泌体回收率高达77.8%,并可以进一步用于人血清等复杂生物液体中外泌体的制备并直接用于下游蛋白质组学分析。尽管该研究在分离效率、成本和时间上相较于传统UC有显著优势,但在处理生物样品时需要将血清等样本进行稀释,此外,仍然存在膜堵塞问题,这在高通量制备外泌体方面存在一定难题。

**图2:**
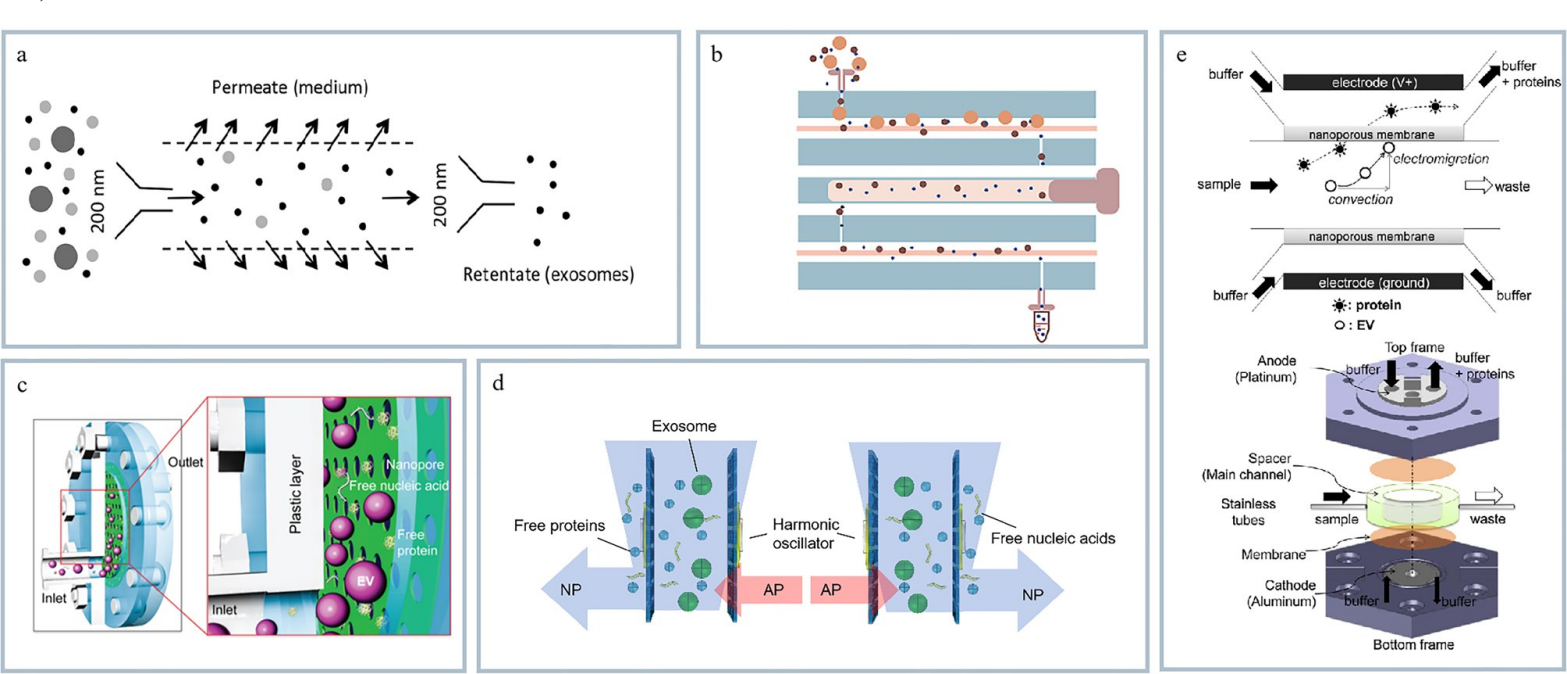
基于外泌体尺寸的膜分离技术

超滤法是利用半透膜的微孔结构以一定的外界压力为推动力实现对物质的选择性分离和回收的方法。近年来,Liu等^[40]^开发了一种高效、快速分离外泌体的纳米孔滤膜芯片(exosome total isolation chip, ExoTIC)(见图2c)。该方法利用目标物的尺寸差异将样品中的杂蛋白、细胞碎片等非外泌体成分有效去除,外泌体被ExoTIC芯片选择性保留,因此显著提高了外泌体的纯度。该方法能够从少量样本中高效分离外泌体,其分离效率远高于传统的UC和聚乙二醇(PEG)沉淀方法。虽然此方法有效提高了外泌体的纯度,但单一方向的超滤技术可能存在膜堵塞的问题,在高通量分离制备外泌体方面仍存在局限性。Chen等^[41]^在ExoTIC基础上开发了一种新型的外泌体超滤策略,通过双向施加压力驱动样品在腔室内运动,基于样品尺寸差异实现外泌体与蛋白质分离(见图2d)。同时结合压电效应使膜发生形变,实现膜的无堵塞以及超快速的外泌体纯化制备。该方法可以在几分钟内处理多种生物液体,并高效、高纯度地分离制备外泌体,避免了高剪切力对样本的破坏问题,显著提高了外泌体的回收率和完整性。进一步通过改变膜的孔径(20、100和200 nm)成功分离制备了3个细胞外囊泡亚群,尺寸区间分别为20~100 nm、100~200 nm和200~450 nm。此外,Cho等^[42]^开发了一种通过电渗和电泳作用分离血浆中EVs的微流体系统(见图2e)。该方法实现了高效率和高纯度的EVs分离,为EVs的分离和研究提供了一种新的技术手段。综上,通过膜过滤的外泌体分离方法具有可扩展性,可以通过改变膜的孔径实现对外泌体以及外泌体亚群的分离制备。

#### 1.1.2 确定性侧向位移

确定性侧向位移(deterministic lateral displacement, DLD)是一种连续流微流控颗粒分离方法。该分离方法通过设计特定的微结构使流体在流动时产生不同的侧向位移实现对不同大小或形状粒子的分离。Wunsch等^[43]^成功开发了纳米DLD阵列并将该技术应用于纳米尺度物质的分离(见图3a)。较大的囊泡被纳米DLD阵列转移到通道的右侧并在右侧通道处收集,而较小的囊泡则遵循Z字形模式流出阵列进入其他通道。该方法可以实现20~110 nm范围内不同尺寸粒子的精准分离,为液体活检提供了新型的微流控分离方法。然而由于该装置需要复杂的精密结构加工,对操作者提出较大挑战。此外,DLD的通量仍然有待进一步提高。

**图3:**
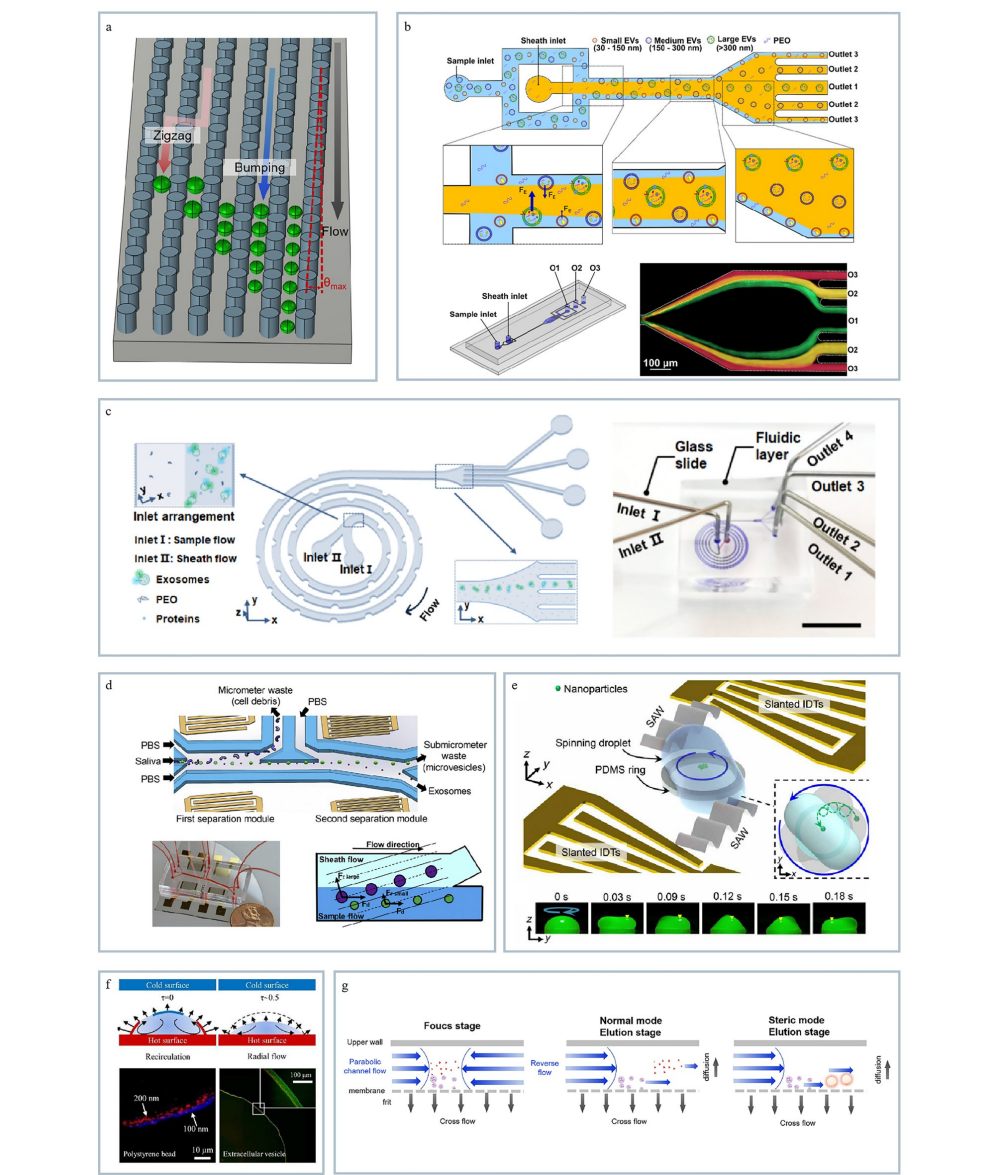
基于外泌体尺寸分离的其他微流控分离技术

#### 1.1.3 黏弹性流体分离

黏弹性微流体技术依赖于非牛顿流体在不同流动环境下的变形特性,实现对颗粒与细胞的精准操控。该技术结合了微流控技术的优势,旨在实现外泌体样品的高通量、高精度处理。Hettiarachchi等^[44]^开发了一种基于黏弹性微流控的新型分离方法,该方法能够实现100~500 nm范围内不同尺寸颗粒的高效分离(见图3b)。通过调整流速比和聚乙烯氧化物(polyethylene oxide, PEO)浓度优化了颗粒在通道中的横向迁移行为,使不同大小的颗粒被高效分离。该装置成功应用于100、200和500 nm三元颗粒混合物的分离,分离纯度最高达到90%,回收率为86%。

#### 1.1.4 惯性微流控

惯性微流体(inertial microfluidics, IMF)是一种利用有限雷诺数下引起的流体动力学效应来被动操控颗粒或细胞的技术。在流体通道中颗粒受到剪切梯度升力和壁面升力等力的作用会在特定位置聚焦或分离。通过设计微通道几何形状和调整流速可以实现高效分离和操控。Bai等^[45]^开发了IMF微流控平台,与传统的直线通道相比,其螺旋微通道结构提升了黏弹性效应,构建的凹形结构产生迪恩流加速粒子聚焦(见图3c)。该方法能在无外加场条件下简单、高效地从少量样本中纯化外泌体,进一步可以结合适配体标记实现单囊泡水平的表面蛋白分析。然而,该方法对流体要求比较高,需要精确优化流体条件,此外,对于分离不同尺寸的外泌体亚群仍然存在一定的局限性。

#### 1.1.5 声波微流控

声波微流控是利用非标记及非接触式的声波来分离和富集外泌体的技术。根据外泌体的大小和密度等差异在声波产生的压力波动和流动场中的运动行为差异实现分离。该方法可以实现外泌体的无损、高效分离,同时可以保持外泌体的生物活性。目前,该技术已广泛应用于纳米颗粒、病毒、单细胞、外泌体等领域。近年来,Wang等^[46]^开发了一种基于声流控技术的唾液外泌体分离方法,该方法能够从未经稀释的唾液样本中高效、高纯度地分离外泌体(见图3d),为液体活检提供了一种新的策略。Gu等^[47]^开发了一种基于声流体学的微流体芯片,实现了在连续流动条件下对纳米颗粒的高效分离(见图3e)。该技术是利用表面声波在微流控芯片中产生微涡旋,通过调整声波频率和流速实现对不同大小纳米颗粒的选择性捕获和富集。通过构建具有双旋转液滴的声流体离心系统实现高纯度的分离纳米颗粒。这种方法不仅提高了外泌体的分离效率,还避免了传统离心分离可能对颗粒造成的损伤问题,为生物医学研究和临床诊断提供了一种新的纳米颗粒分离平台。尽管该方法可以分离外泌体以及外泌体亚群,但在处理复杂的生物样品时非外泌体成分可能影响灵敏度和分离效果。

#### 1.1.6 其他分离方法

Jeong等^[48]^利用液滴蒸发过程中的咖啡环效应实现了对EVs的尺寸分离(见图3f)。通过控制液滴蒸发过程中的温度梯度调整液滴边缘的接触线动态,使不同尺寸的细胞外囊泡在干燥环中形成分离的环状图案。较小的细胞外囊泡倾向于靠近接触线沉积,而较大的细胞外囊泡则在液滴中心附近聚集。此外,该研究还发现细胞外囊泡的尺寸与其表面蛋白表达水平相关,为细胞外囊泡的进一步研究和应用提供了新的分离策略和理论依据。然而该技术目前仍存在外泌体的分离制备通量低、难以回收外泌体等技术瓶颈,限制了进一步临床应用。

非对称流场流分离技术(asymmetric flow field-flow fractionation, AF4)是一种用于样品分析和半制备的技术,其主要利用流场和外场结合实现颗粒分离。在膜分隔的通道内流动时,样品因其大小和形状差异而受到不同程度的作用力,进而实现高分辨率的分离。该方法适用性强且对样品损伤小,可用于样品的半制备。Zhang等^[49]^将AF4技术用于分离和表征工程化的纳米颗粒(engineered nanoparticles, ENPs),包括小细胞外囊泡(sEVs,即外泌体)和更小的外泌颗粒(exomeres)(见图3g)。与传统技术相比,AF4技术具有无需标记、操作温和、快速(<1 h)、高重复性等优点。此外,AF4技术能够实现高分辨率分离,这对于外泌体和外泌颗粒的分子诊断和临床治疗具有重要意义。然而,目前AF4技术需要专业操作人员才可以实现目标物的分离,因此该技术的通用性以及普适性有待提高。

### 1.2 基于外泌体电荷分离的微流控技术

外泌体双层膜结构中的磷脂和糖蛋白等成分使外泌体表面通常带有负电荷。带负电荷的外泌体在生物体内和体外环境中能够与细胞、蛋白质发生多种相互作用。微流控技术可以基于外泌体的带电特征在微通道内整合电泳等原理实现外泌体分离,分离过程通常比传统方法更迅速,对外泌体的结构和功能影响较小,有利于后续分析。

#### 1.2.1 凝胶电泳

凝胶电泳是一种根据目标物的质荷比在多孔基质中的电迁移行为不同实现分离的技术。传统的凝胶基质有琼脂糖、聚丙烯酰胺等,目前凝胶电泳技术已广泛应用于蛋白质、DNA等生物大分子的分离^[50,51]^。在多孔凝胶基质中,电荷尺寸比大的目标物在凝胶中迁移得更快,电荷尺寸比小的目标物则迁移得较慢^[52]^。基于以上原理,Zhang等^[53]^探究了琼脂糖凝胶电泳技术分离外泌体的可行性,通过优化pH值和琼脂糖凝胶浓度对外泌体迁移速度的影响,实现了从血浆中高效分离外泌体和脂蛋白(见图4a)。该方法无需标记,分离时间短,但从凝胶基质中回收外泌体时需要多步样品处理过程,难以保证外泌体的活性和完整性。

**图4:**
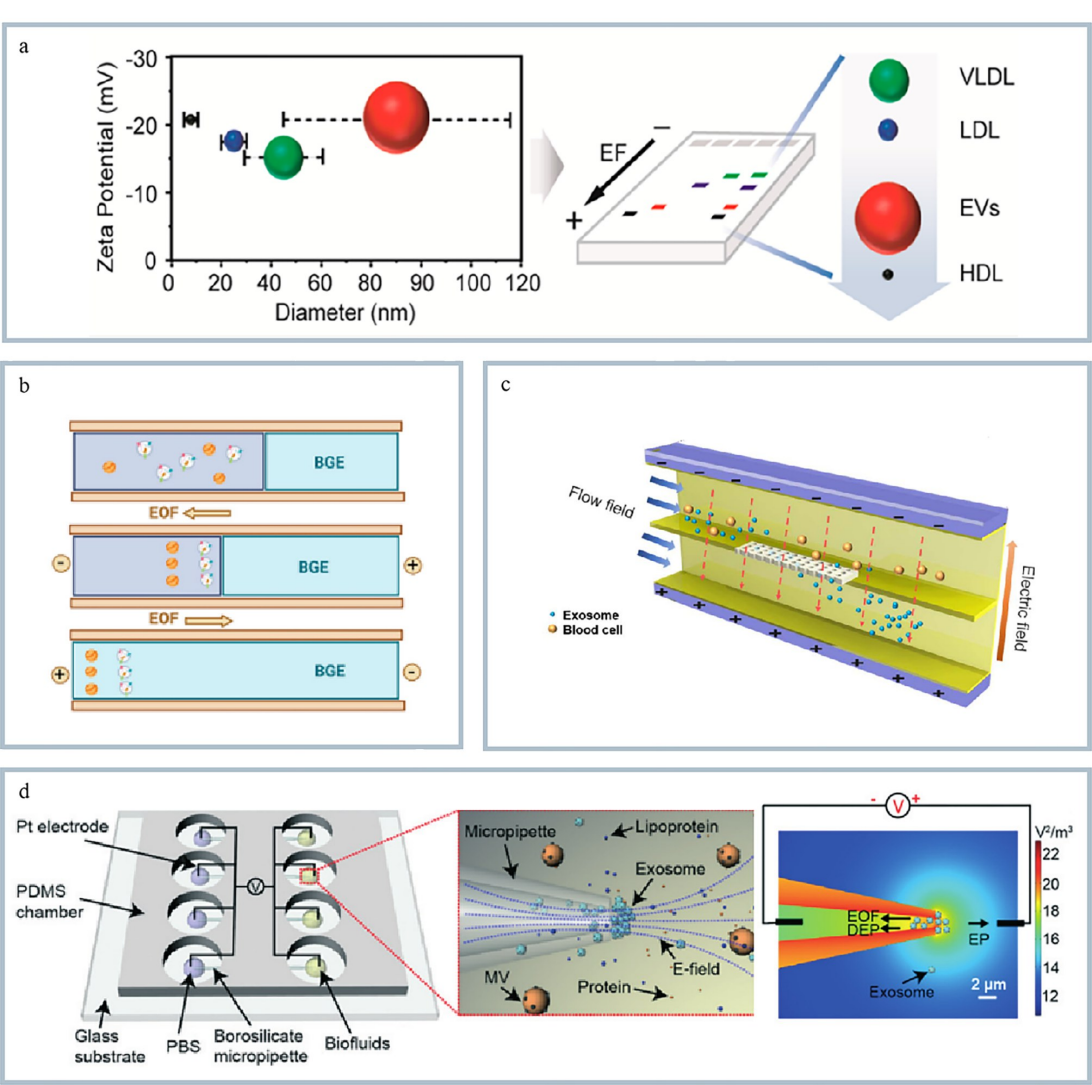
基于外泌体的表面电荷的分离技术

#### 1.2.2 毛细管电泳

毛细管电泳是近年来发展最快的分析方法之一,它是以毛细管为分离通道,以高压直流电场为驱动力形成定向运动的电渗流,通过电渗以及电泳的作用实现目标物的分离。Gao等^[54]^将大体积样本堆叠技术(large-volume sample stacking, LVSS)与毛细管电泳相结合(见图4b),并与非对称流场流分离系统联用提高了从血清中分离细胞外囊泡的效率和纯度。该方法通过优化样本处理和分离条件显著减少了非蛋白成分的干扰,提高了EVs的回收率和蛋白鉴定的准确性,为EVs的深入研究和临床应用提供了新的技术平台。

#### 1.2.3 自由流电泳

FFE是一种半制备电泳分离技术,目前已广泛用于分离细胞、蛋白质和外泌体等样品。在FFE中被分离物质受到垂直于液流方向的电场力作用,样品中各种组分由于各自电泳迁移率的差异而各自向与所带电荷相反的电极方向迁移,相同电泳迁移率的物质则迁移为一窄带,在到达分离腔出口处由收集器收集。FFE可以实现在线、连续分离,对样品损伤小以及分辨率高。Hu等^[55]^开发了一种结合流体和电场的微流控平台用于高效分离外泌体(见图4c),该平台通过模拟和实验验证外泌体的分离效果,其回收率高达84.2%,为外泌体的分离提供了一种新方法。

#### 1.2.4 介电泳技术

DEP是一种基于在非均匀电场中颗粒介电特性差异从而实现目标物分离和富集的技术。DEP具有非接触操控、高选择性、快速、高效等优点,目前该方法已经广泛应用于细胞、纳米颗粒、外泌体等的分离富集。近年来,Shi等^[36]^开发了一种新型DEP设备,能够在20 min内从200 μL样本中快速富集小细胞外囊泡(见图4d)。与传统的差速离心法相比,通过该方法富集的纳米囊泡浓度提高两个数量级,且样本制备简单,产量高,对囊泡形态和完整性影响小,尤其在复杂样品中分离富集外泌体方面具有较好的效果;因此DEP技术有望成为临床液体活检中快速分离小细胞外囊泡的强有力工具。然而,该技术仍存在设备复杂、通量低等局限性,在面临实际应用时需优化出精确的实验条件,因此限制了其临床转化。

### 1.3 基于外泌体免疫亲和捕获的微流控技术

免疫亲和是指利用特异性抗体与目标分子之间的相互作用来实现分离和富集的一种方法。在外泌体分离中,通过将外泌体表面特定蛋白如 CD63、CD9、CD81的抗体固定在分离介质(如微流控芯片、磁珠)上,从而高效捕获目标外泌体,同时去除未结合的成分,实现外泌体的高效分离和分析。近年来基于外泌体表面识别多肽^[56]^和适配体^[57]^等的方法逐渐涌现,为外泌体的分离和纯化提供了新途径。

#### 1.3.1 免疫捕获

Chinnappan等^[58]^开发了一种集成化的微流控芯片平台用于结直肠癌外泌体的预浓缩和检测(见图5a)。该方法是利用抗CD63适配体作为识别元件,通过免疫磁珠技术实现了外泌体的高效捕获和富集。Hong等^[59]^开发了一种基于楔形结构和高磁场梯度介导芯片的方法检测口腔溃疡和口腔癌来源的外泌体。该方法可以选择性地修饰抗体用来区分口腔癌和口腔溃疡的外泌体,显著提高了诊断的准确性。同时该方法具有较高的灵敏度和特异性,外泌体的检出限低至8.5×10^4^个/mL,为早期口腔癌和口腔溃疡的鉴别及临床诊断提供了新策略。

**图5:**
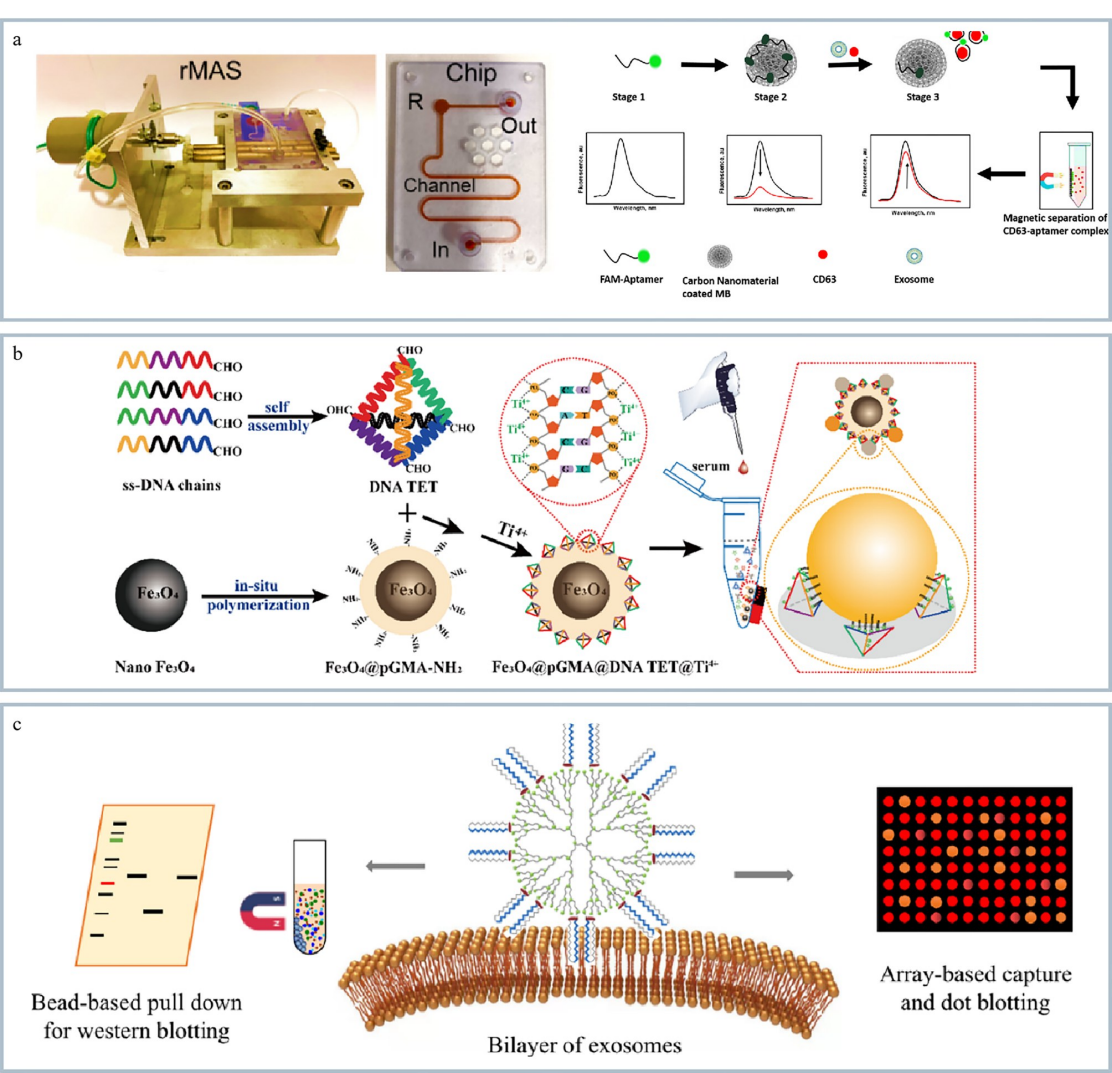
基于外泌体的免疫亲和捕获的分离技术

#### 1.3.2 亲和捕获

近年来,Chu等^[60]^开发了一种可编程框架核酸修饰的纳米磁珠(Fe_3_O_4_@PGMA@DNA TET@Ti^4+^)用于高效分离外泌体(见图5b)。这种纳米磁珠与外泌体双层膜结构上的磷酸基团结合,通过特定的DNA四面体结构(DNA TET)和钛离子(Ti^4+^)的协同作用实现了对外泌体的高特异性捕获和快速释放,有效提高了外泌体的分离效率。进一步通过外泌体蛋白质组学分析挖掘生物标志物,为外泌体研究和临床诊断提供了新的技术平台。

#### 1.3.3 两亲性超分子探针

随着研究的不断深入,基于外泌体的双层膜与两亲性超分子探针之间的多价相互作用可以实现外泌体的高效分离捕获。Feng等^[61]^开发了首个基于超分子探针同时捕获和分析外泌体的方法。该方法通过将两性霉素B(amphotericin B, AMB)与三维树枝状大分子偶联显著增强了与磷脂膜的相互作用,可以从复杂体液中有效地捕获外泌体(见图5c)。该方法进一步应用于临床样品肝癌患者和健康对照组的血浆样品中外泌体的捕获,结果发现该方法检测灵敏度高且通量高,为临床外泌体的分析和肿瘤的早期诊断提供了新的分子工具。

综上,基于微流控的外泌体分离技术见表1。

**表1:** 基于微流控的外泌体分离技术

Microfluidic technology	Advantages	Limitations	Samples	Sample volume	Isolated size/nm	Time/ min	Recovery/ %	Ref.
Exosome separation based on size								
TFF	high throughput, con- tinuous preparation	complex operation, lengthy duration	cell culture super- natant, plasma	6 mL	0-350	200	77.80	[39]
ExoTIC	high recovery rate, easy to operate, short time consuming	low processing volume	cell culture super- natant, urine, plasma	10 mL	30-100	125	90	[40]
EXODUS	high recovery rate, easy to operate, short time consuming	low processing volume, complex equipment	cell culture super- natant, urine, tears, plasma	10 mL	30-200	10	100	[41]
Electrophoresis- driven filtration	easy to operate, short time consuming	low processing volume	plasma	1 mL	10-400	30	65	[42]
Nano-DLD pillar arrays	minimal damage	low processing volume, complex operation, lengthy duration	urine	10 μL	20-110	50000	NA	[43]
Viscoelastic fluid separation	high recovery	complex operation, lengthy duration	cell culture supernatant	1 mL	30-500	50	86	[44]
Inertial microflu- idics	minimal damage	low processing volume, complex operation, lengthy duration	cell culture supernatant	1 mL	0-400	100	70.60	[45]
Acoustofluidic technology	minimal damage	complex operation	saliva	NA	30-150	20	75	[46]
Acoustofluidic centrifuge	high recovery rate, short time consuming	low processing volume, complex operation, lengthy duration	plasma	80 μL	30-150	<1	>80	[47]
Coffee ring effect	minimal damage	cannot be collected	cell culture supernatant	NA	100-200	NA	NA	[48]
AF4	minimal damage	complex operation, lengthy duration	cell culture supernatant	NA	35-150	50	NA	[49]
Exosome separation based on surface charge								
Plate gel electro- phoresis	high recovery rate, minimal damage, easy to operate	difficulty in recycling, low processing volume, lengthy duration	urine	20 μL	10-250	120	100	[53]
Capillary electro- phoresis	minimal damage	low processing volume, complex operation, lengthy duration	plasma	10 μL	100-200	26	75	[54]
Free field electro- phoresis	high recovery rate, minimal damage	low processing volume, complex operation, lengthy duration	pure exosome solution	1 mL	35-150	100	84.2	[55]
Dielectrophoresis	short time consuming	low processing volume, complex operation	plasma, saliva	200 μL	80-200	20	NA	[36]
Exosome separation based on immunoaffinity								
Integrated lab-on- a-chip platform	high recovery rate, high specificity	complex operation, lengthy duration	cell culture supernatant	10 mL	0-150	2000	90	[58]
Wedge high mag- netic field gradi- ent mediates chip	high recovery rate, high specificity	complex operation, long time consuming, low processing volume	cell culture supernatant	50 μL	0-400	10	NA	[59]
IMAC nanosized magnetic	high recovery rate, high specificity	low processing volume	cell culture super- natant, plasma	1 mL	50-500	5	90	[60]
Supramolecular exosome array	high recovery rate, high specificity	low processing volume	saliva, urine, plasma	10 μL	30-140	40	71	[61]

TFF: tangential flow filtration; NA: not available.

## 2 基于微流控的外泌体分析技术

外泌体的来源异质性、尺寸异质性决定了其内含物异质性和生物学功能异质性,因此如何分析异质性外泌体并深度解析外泌体的特异性功能及其对疾病发生过程的作用机制是需要解决的关键科学问题。传统的外泌体分析技术有透射电子显微镜^[62]^、纳米颗粒追踪分析^[63,64]^、动态光散射^[65,66]^、酶联免疫吸附试验^[36]^、蛋白免疫印迹^[67⇓-69]^、质谱^[70⇓⇓-73]^等技术,这些技术通过评价外泌体的浓度、尺寸、形貌、特异性生物标志物的表达情况等对全尺寸外泌体或外泌体亚群进行分析。然而,这些分析手段检测复杂体液中的外泌体时难以避免生物分子的非特异性吸附问题。同时,这些技术均是针对外泌体群体进行分析,然而外泌体存在异质性,难以精密解析外泌体功能异质性的内涵。而微流控技术因样品消耗量以及试剂消耗量少、集成性、可操作性、便携性等优势在外泌体分析方面具有极大优势。在微型化的微流控芯片上,通过集成化系统实现样本进样、目标分离与信号检测的全流程整合,该技术体系在外泌体生物标志物的临床鉴定以及肿瘤等重大疾病的早期诊断中展现出显著的应用潜力。同时,基于微流控技术也可以实现在单个外泌体水平上对特定分子如蛋白质、核酸、代谢物等进行高灵敏检测和快速分析,用以揭示外泌体在异质性和功能特性上的细微差异。基于微流控检测方法的不同,将基于微流控的外泌体分析技术大致分为电化学检测、磁偏转检测、比色法检测、光学检测等几大类,详见表2。

**表2:** 基于微流控的外泌体分析技术

Microfluidics	Principles	Advantages	Limitations	Analysis objects	Ref.
Electrochemical detection					
MOF-functionalized sensing	a MOF-based sensing interface for exosome capture and an enzyme- based logical gate for signal trans- duction and data processing	high selectivity	easy to be disturbed	surface marker, nucleic acid molecule	[74]
Nanopipette-assisted method	the amperometric device measures the electrochemical redox peaks generated during the release of DA from a single exosome	high sensitivity	complex operation	metabolites	[75]
Magnetic deflection detection					
Magnetically driven nanomechanical sensors	magnetic fields deflect magnetic materials that bind exosomes	high sensitivity, high selectivity	complex operation	surface marker	[76]
Colorimetric detection					
Colorimetric aptasensor	exosomes interact with colorimetric reagents, resulting in color changes in the solution	high sensitivity, high selectivity	easy to be disturbed	surface marker, nucleic acid mole- cule, metabolites	[77]
DNAzyme-RCA-based colorimetric and lateral flow dipstick assays	exosomes interact with colorimetric reagents, resulting in color changes in the solution	high selectivity, portable	complex operation	surface marker, nucleic acid mole- cule, metabolites	[78]
Optical detection					
Exosome isolation and detection system	fluorescent probe to label exosomes	immune capture	single object	surface marker	[79]
Microfluidic surface- enhanced Raman scattering sensor	rolling circle amplification and tyramine signal amplification	high sensitivity	complex operation	nucleic acid molecule	[80]
Nanoplasmonic pillars	local surface plasmon resonance is realized by a gold nanosensor matched to the size of a single exosome	individually imaged in real time, high sensitivity	easy to be disturbed, complex operation	surface marker	[81]
Frequency-locked microtoroid optical resonators	changes in the resonant frequency of the microtoroid	high sensitivity	high requirements on samples	size, mass, polarizability	[82]

MOF: metal-organic framework; DA: dopamine; RCA: rolling circle amplification.

### 2.1 电化学检测

电化学技术具有成本低、样品量少、灵敏度高的特点,在微流控方法中被广泛用于外泌体的分析检测。该方法利用外泌体与修饰电极表面的相互作用,通过特定电化学信号的变化实现快速以及高灵敏检测。Wang等^[74]^构建了一种多功能传感界面可直接从生物流体中检测外泌体(见图6a),通过单芯片传感策略可以同时检测外泌体蛋白和RNA标志物。该方法引入了酶基逻辑门的概念,克服了外泌体异质性的难题,成功地识别了乳腺癌患者来源外泌体,其灵敏度和特异性均接近100%,为肿瘤早期诊断提供了新思路。

**图6:**
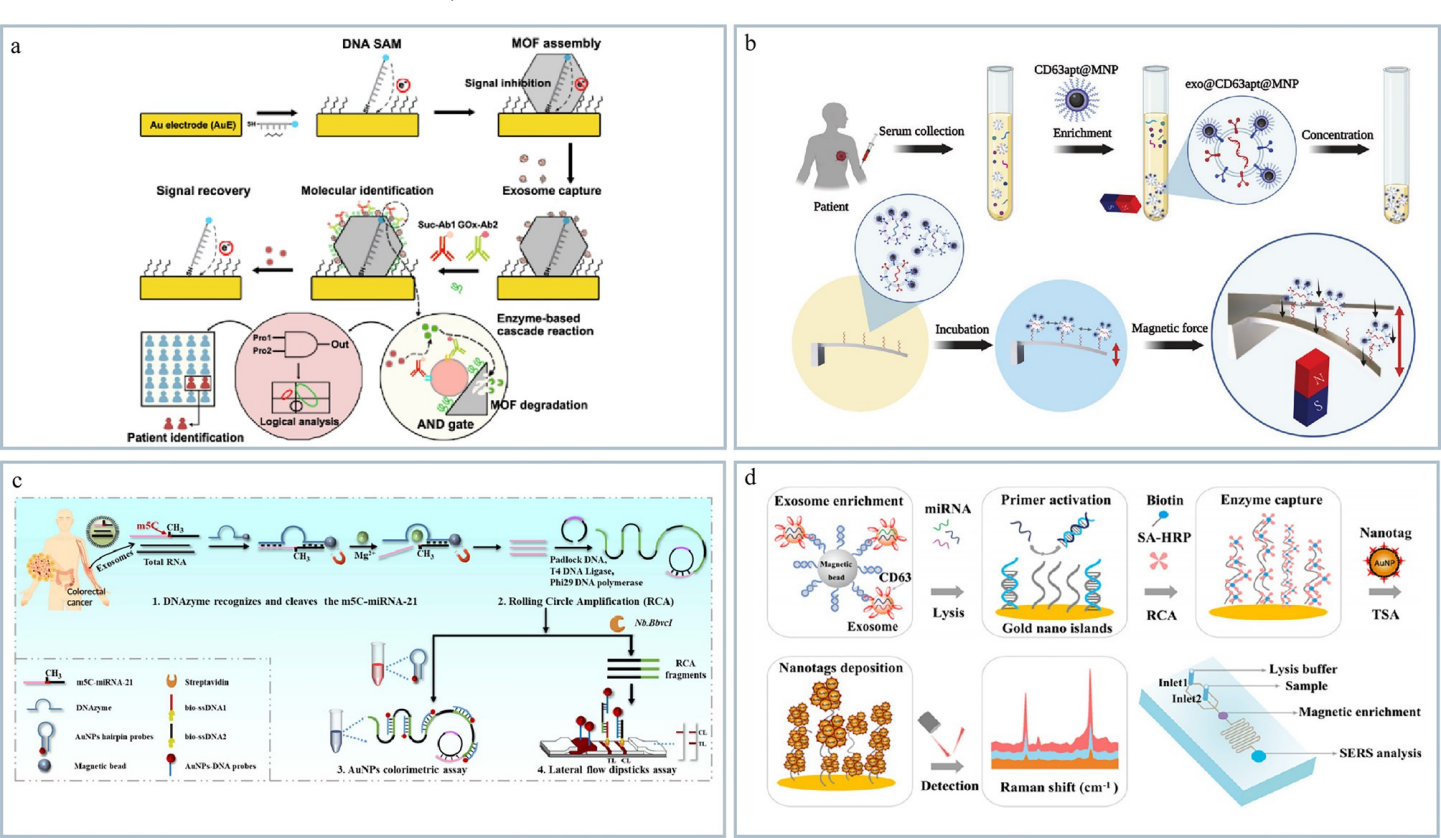
基于微流控的外泌体分析技术

除此之外,基于微流控技术的电化学检测也可以捕捉单个外泌体的电化学信号,实现单个外泌体的实时动态检测。其中基于纳米孔的单个外泌体传感技术是通过检测纳米孔中单个外泌体通过时的电流变化来分析外泌体大小、电荷或内容物。Lv等^[75]^通过在针尖内壁喷涂超薄的金膜制备了一种金膜沉积纳米管,利用安培测量法检测单个外泌体及其多巴胺(dopamine, DA)含量。该方法可同时监测外泌体转运和检测单个外泌体的DA含量信息,并构建了机器学习模型,其准确率可达99%。本方法有助于研究含DA外泌体的快速检测,为相关外泌体研究提供了新方法。

### 2.2 磁偏转检测

磁偏转技术是基于外部施加的磁场引起磁性材料(如磁珠或磁性纳米颗粒)发生偏转,从而检测外泌体与磁性材料结合后的信号的一种检测方法。该方法具有高灵敏度和高特异性的特点,适用于复杂生物样本中外泌体的分析。Mei等^[76]^开发了一种磁驱动的微悬臂阵列,可选择性地检测致癌外泌体(见图6b)。通过将CD63适配体和表皮生长因子受体(epidermal growth factor receptor, EGFR)适配体分别作为MDA-MB-231细胞分泌的外泌体捕获和识别探针,构建了磁驱动纳米机械传感器,将外泌体检出限降低至小于10个/mL,该方法的检出限比传统检测方法相比降低了5个数量级,极大地提高了外泌体的检测灵敏度。

### 2.3 比色法检测

比色法检测是利用外泌体与比色反应试剂(如纳米颗粒、酶底物)之间的特异性相互作用导致溶液颜色变化,通过光谱或肉眼观察颜色变化实现外泌体的检测。这类方法具有操作简单、无需复杂设备、速度快、成本低的优势,通过颜色变化即可定性定量分析外泌体,适合外泌体的快速筛查。Xu等^[77]^利用辣根过氧化物酶(HRP)加速无色的DA聚合和棕黑色的聚多巴胺(polydopamine, PDA)原位沉积,实现对癌症来源外泌体的高灵敏、特异性检测。Zhang等^[78]^开发了基于DNAzyme-RCA(rolling circle amplification, RCA)的金纳米粒子比色法和侧向流试纸条检测法用于外泌体m5C-miRNA-21的即时检测(见图6c)。该方法能特异性识别和线性扩增目标物,通过金纳米粒子聚集实现低背景干扰颜色输出,具有操作简便、成本低的优势,为结直肠癌等相关疾病的诊断提供了新的即时检测策略。但比色法的灵敏度和定量精度较低,易受背景干扰,因此限制了其临床应用。

### 2.4 光学检测

在众多基于微流控技术的外泌体分析手段中,光学检测是一种最常用的检测手段,包括荧光检测、表面增强拉曼(surface-enhanced Raman scatting, SERS)、表面等离子共振等方法。其中,荧光成像技术因具有精度高、灵敏度高等优点已广泛用于微流控芯片的外泌体检测。Lu等^[79]^提出了集成式微流控(exosome isolation and detection, EXID)系统用于实现外泌体的分离和生物标志物检测。该方法将分析时间降至2 h以下,能实现对单个磁珠进行分析并且同时检测外泌体表面蛋白的异质表达水平。同时,通过该系统可分析多种细胞系和临床样本中外泌体PD-L1的表达,为肿瘤个性化诊断和免疫治疗提供了有潜力的工具。表面增强拉曼是一种利用金属纳米颗粒显著增强拉曼信号的共振光学检测技术。当分子靠近金属表面时,局部电场的增强使得拉曼散射信号显著提高。SERS具有高灵敏度、快速分析、无标记检测的特点,因此也是目前比较常用的外泌体检测手段之一。Zhao等^[80]^构建了基于核酸-酪胺级联放大的微流控SERS传感器用于外泌体miRNA分析(见图6d)。该方法整合了外泌体富集、裂解和miRNA检测于一体,避免样本消耗过大的问题,提高定量准确性。通过引入RCA和酪胺信号放大(tyramine signal amplification, TSA)策略,使目标miRNA被扩增产生大量催化单元,大量纳米标签沉积于SERS底物上实现超高灵敏度检测,结果显示该方法对外泌体的检出限可以低至1 pmol/L。

共振光学检测技术在单个外泌体高灵敏度、高选择性和无标记检测等方面独具优势。Raghu等^[81]^开发了用于外泌体检测的局部表面等离子体共振成像技术(localized surface plasmon resonance imaging, LSPRi),通过设计金纳米传感器的尺寸,使其与外泌体尺寸相近,实现了单个外泌体的数字化响应,并通过将传感元件置于石英柱上减少了底物非特异性吸附的影响,实现高通量数据收集,为实时检测活细胞分泌的外泌体提供了可能。Su等^[82]^利用微环光学谐振器实现了血清中单个外泌体的无标记、实时检测。该方法利用光在微米级别的环形结构中传播形成的谐振现象,当外泌体进入微环的渐逝场时会改变微环的有效折射率,从而使微环的共振频率发生偏移。通过将测量到的信号变化与已有理论相结合,实现对结合外泌体的大小、质量和极化率的估算,从而获取肿瘤的相关信息,为肿瘤活检提供依据。

## 3 疾病诊断及临床应用

外泌体作为细胞间通讯的重要媒介携带了大量的分子信息,在疾病诊断和临床治疗中具有重要价值,可以作为液体活检的一种有效手段。外泌体中携带的蛋白质^[83]^、RNA^[84]^和脂质^[85]^等内含物可以作为癌症、神经性疾病和心血管疾病等的生物标志物。微流控技术在外泌体疾病诊断和临床应用中具有显著优势^[86]^,该平台能够高效地处理多个临床样本并实现疾病来源外泌体的高通量、快速、精准分析。

### 3.1 疾病诊断

体液(血液、泪液、尿液、唾液等)中富含多种来源外泌体,其收集过程相对简单,属于非侵入性手段。通过微流控技术可以直接进样分离并检测体液中的外泌体样本,为疾病诊断提供有效手段。Kim等^[87]^开发了一个无标记的miRNA传感平台(见图7a),利用三维分层等离子体纳米结构的表面增强拉曼散射检测尿外泌体miRNA差异表达水平可以准确区分前列腺癌患者和健康对照组。Yin等^[88]^开发了一种基于石墨烯的传感器阵列,该方法是利用场效应晶体管技术(graphene field effect transistor, GFET)通过特异性抗体捕获外泌体,并通过电信号变化成功地检测到与胰腺导管腺癌(pancreatic ductal adenocarcinoma, PDAC)相关的特定外泌体标志物,即GPC-1抗体(见图7b)。该方法能够在稀释的血浆样本中准确识别出PDAC患者和健康对照组之间的差异。Hu等^[89]^开发了一种眼泪外泌体的快速分离分析系统(iTEARS),能够从泪液中快速分离并分析外泌体(见图7c)。基于该方法中的纳米多孔膜共振器可以实现从几滴泪液(约10 μL)中快速高效地分离外泌体,通过蛋白质组学和转录组学分析进行定量检测和识别生物标志物,共鉴定出904种蛋白质,其中426种蛋白质如CALML5、KRT6A和S100P可用于眼干燥症的分类。因此微流控技术在外泌体检测的临床应用中具有实用价值。随着研究的深入,微流控技术在外泌体检测领域有望发现新的生物标志物和疾病机制,推动个性化医疗的发展。

**图7:**
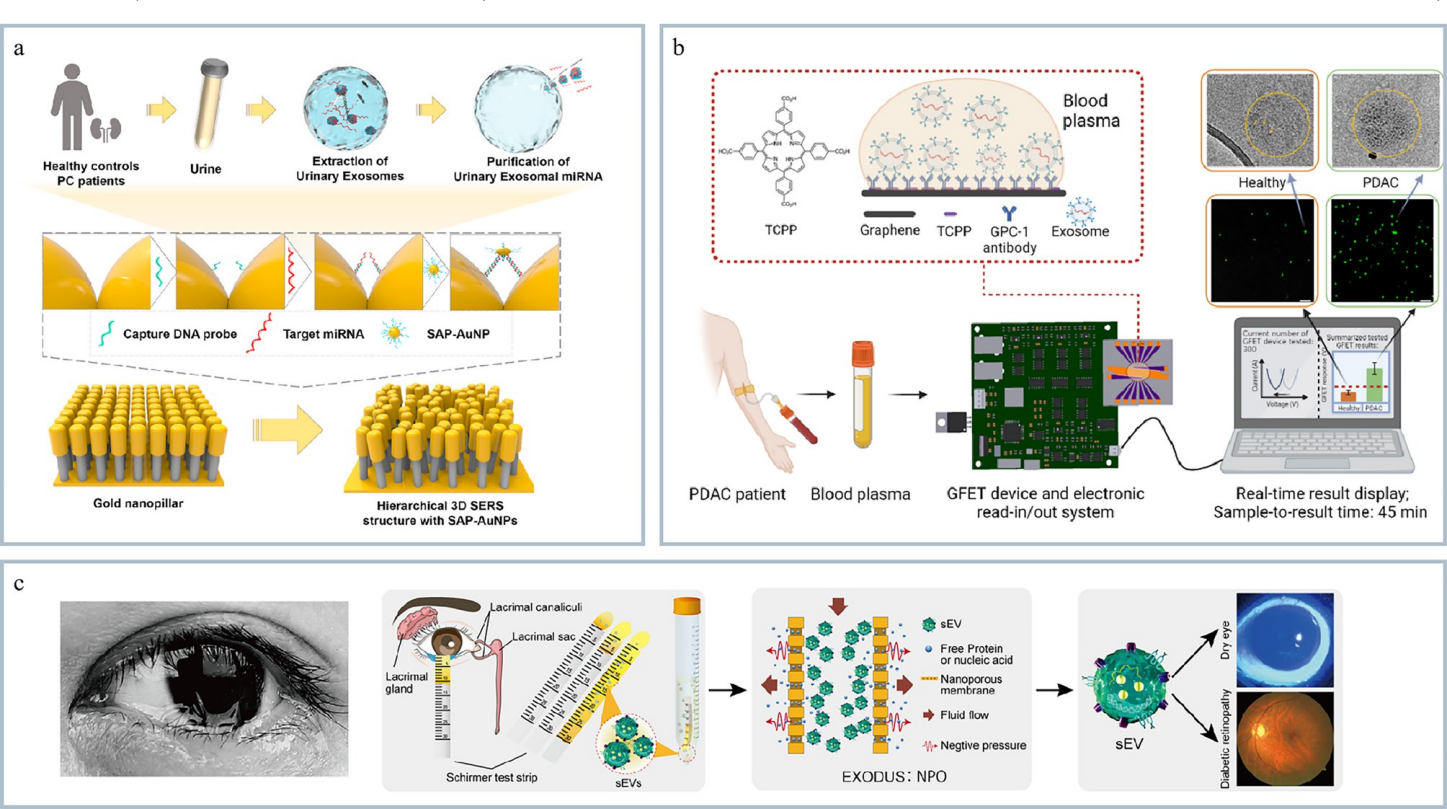
外泌体的疾病诊断及临床应用

### 3.2 疾病治疗

外泌体具有优良的生物相容性和低免疫原性,其脂质双层结构使其能够跨越细胞屏障并将携带的蛋白质、miRNA和其他生物活性分子靶向递送至目标部位^[90⇓⇓⇓-94]^。作为一种新型的细胞间通讯及信息传递的载体,目前外泌体在肿瘤治疗、神经退行性疾病、心血管疾病和再生医学等领域展现出广阔的应用潜力。例如,Zhu等^[95]^利用脐带间充质干细胞来源的外泌体(UC-MSC-Exo)促进皮肤和神经的再生。该技术不仅在体外验证了UC-MSC-Exo对成纤维细胞增殖和神经生长因子分泌的促进作用,进一步通过动物模型证实了在皮肤切除模型中加速伤口愈合的能力,还探讨了UC-MSC-Exo在改善皮肤神经周围环境、促进神经再生方面的潜力,为外泌体在神经修复中的临床应用提供了新的视角。此外,为了提高外泌体的功能性和靶向性,可以通过工程化外泌体对天然外泌体进行化学修饰或基因修饰,进而有效控制外泌体的尺寸、表面修饰以及内容物^[96⇓-98]^。Ding等^[99]^利用源自人间充质干细胞(hUCMSCs)的外泌体传递外源性miR-145-5p。研究结果表明这种外泌体能够有效抑制PDAC细胞的增殖和侵袭,增强细胞凋亡和细胞周期阻滞。通过体内外实验证实了外泌体在肿瘤治疗中的潜力,并为PDAC的治疗提供了新策略^[8]^。尽管上述外泌体在疾病治疗方面展现出巨大潜力,但如何进一步优化控制天然外泌体以及工程化外泌体的制备和检测方法,提高其安全性、有效性和稳定性是亟待解决的问题。作为新型疾病治疗策略,相信外泌体将发挥越来越重要的作用。

## 4 结论与展望

外泌体的精准分离、制备、检测及功能研究是目前分析化学和生物医学领域的研究前沿和热点。作为体内的一种“物联网、互联网”物质,外泌体在生物体内信息传递、疾病机理和诊断、生物治疗等研究领域中起着重要作用。然而如何从复杂的生物样品中精准分离且获取外泌体的生物信息为外泌体研究带来了极大挑战。本文主要针对外泌体的尺寸、电荷、表面官能团等特征,结合基于微流控技术功能进行了综述,重点介绍了基于微流控技术实现外泌体尺寸分离、电荷分离以及表面抗体及适配体等的分离方法;此外,基于微流控技术的通量、集成化、试剂消耗少等特征详细阐述了外泌体群体分析方法及单个外泌体分析方法,为疾病诊断及治疗提供了一个全新的分离平台。

尽管微流控技术在外泌体的分离与分析上取得了显著突破,外泌体的来源异质性和尺寸异质性决定了其内含物的异质性及生物学功能的多样性。因此,如何有效分离异质性外泌体并深入解析其特异性功能及对疾病发生机制的作用仍然是亟待解决的关键科学问题。外泌体的异质性不仅体现在不同来源的细胞之间,还体现在同一来源的外泌体在不同外部刺激或状态下的内容物差异。这种异质性使得外泌体的生物学信息更加复杂,进而影响其在疾病研究及治疗中的应用。为了探究和阐明外泌体的“量-效”关系,尤其是在生命科学研究中的深远意义,精细尺寸外泌体的分离与分析显得尤为重要。精确分离外泌体亚群有望提高外泌体在疾病治疗中的效果,特别是在癌症、神经退行性疾病等领域。然而,外泌体的异质性也显著限制了其亚群研究的深入,尤其是如何从复杂样本中分离出具有高度特异性和生物学功能的外泌体。现有分离技术往往面临精准度、纯度和产量的权衡,因此亟需兼容高通量、快速、个性化的分析技术。尤其是针对单个外泌体的分析要求更高的灵敏度和准确性。随着新型分离技术、标志物识别手段以及单外泌体分析技术的不断发展,微流控芯片技术有望在精细尺寸外泌体的分离与分析研究中取得更大的突破。这些进展不仅能够提升外泌体在疾病早期诊断、靶向治疗等领域的应用潜力,也为个性化医疗提供了重要的技术支撑。

总之,相信外泌体将在更多疾病的治疗中发挥重要作用,为医学领域带来更多的创新和突破。
